# A comparative study of operative and conservative treatment of intraarticular displaced calcaneal fractures

**DOI:** 10.1038/s41598-021-83636-9

**Published:** 2021-02-17

**Authors:** K. Ramachandra Kamath, Sharan Mallya, Atmananda Hegde

**Affiliations:** Department of Orthopaedics, Kasturba Medical College, Mangalore Campus, Manipal Academy of Higher Education, Manipal, Karnataka 576104 India

**Keywords:** Health care, Medical research

## Abstract

The treatment of intra-articular displaced calcaneal fracture is debatable. We conducted a prospective study to compare operative and non-operative treatment for intra-articular displaced calcaneal fractures. Patients were assigned to two groups based on the treatment given (operative and nonoperative) and were regularly followed for a period of 1 year. The outcome measures were assessed by Modified Rowe’s Score (MRS), Visual Analogue e Scale (VAS) and The American Orthopaedic Foot and Ankle Society (AOFAS) scale. The outcome related to patient’s job was noted after one year and compared with pre-injury status. Fifty five patients with 61 calcaneal fractures were studied. Thirty of them were operated and 31 were treated conservatively. Out of 30 operated cases, Bohler’s angle was restored in 25 cases and these had good results with all three outcome scores at 1 year follow up and remaining 5 cases showed fair results (Mean MRS: 74.783, VAS: 3.348, AOFAS: 78.783). Thirty one cases treated with cast also showed fair results (Mean MRS: 57.368, VAS: 4.944, AOFAS: 71.211). The overall outcome of operated cases were better than non-operated cases (unpaired T test MRS: 5.807 *p* < 0.001, VAS: 4.387 *p* < 0.001, AOFAS: 2.728 *p* = 0.008) . Operative treatment of displaced intra-articular calcaneal fractures gave good results at one year follow up, provided Bohler’s angle was restored to normal range. Non operative treatment gave fair results. Complications were seen both with operative and non-operative treatment.

## Introduction

Calcaneal fractures are commonest of tarsal fractures and approximately 75% of these are intra-articular^1^. Complicated anatomy and poorly understood hind foot kinematics are the foremost difficulties in the management of calcaneal fractures. The bone has very minimal soft tissue cover and very limited amount of dense cortical bone, hence fracture patterns are tremendously varied. All these problems make it difficult to treat calcaneal fractures^2^. Controversy remains with regard to whether displaced intra-articular calcaneal fractures should be treated operatively or conservatively^3^. Historically, displaced intra-articular calcaneal fractures were treated non-operatively as predictable operative reduction and fixation were not possible. Operative reduction became popular as fracture care improved^3^. Open reduction and internal fixation of intra-articular calcaneal fractures can only be expected to benefit those patients in whom nearly anatomical reconstruction is obtained. Operative treatment which does not result in anatomical reconstruction has shown poor outcome^3^. Comminuted displaced fractures, male gender, and heavy manual labor are associated with poor outcome^1^. The optimal management of displaced intra-articular calcaneal fractures remains a matter of debate despite advancements in diagnosis by means of imaging and surgical techniques. Although modern operative intervention has improved the outcome in many patients, there still is no real consensus on, treatment, operative technique or postoperative management^4^.

Our study aimed to compare the functional outcome, quality of life and residual pain following displaced intra-articular calcaneal fractures treated operatively and non-operatively.

## Methodology

An observational study was conducted on fifty-five patients with closed displaced intra-articular calcaneal fractures, aged between 18 and 65 years. The study included patients treated in the Department of Orthopaedics in our institution. Patients with undisplaced fracture (Sanders type 1), extra-articular fractures, comorbidity like diabetes, associated spine fractures with neurological deficits, open fractures were excluded. Informed and written consent was taken from all patients. The study was cleared by the Institutional Ethics Committee (IEC KMC MLR 12-13/276) and all the regulations and guidelines have been followed.

Sixty-one calcaneal fractures were studied. Patients were assigned to two groups (operative and non-operative). Randomization was done based on alternate allocation. X-ray and CT scan was done for all patients. Using a CT scan, fractures were classified as per Sanders classification^4^ type II, III, IV.

**Figure 1:**
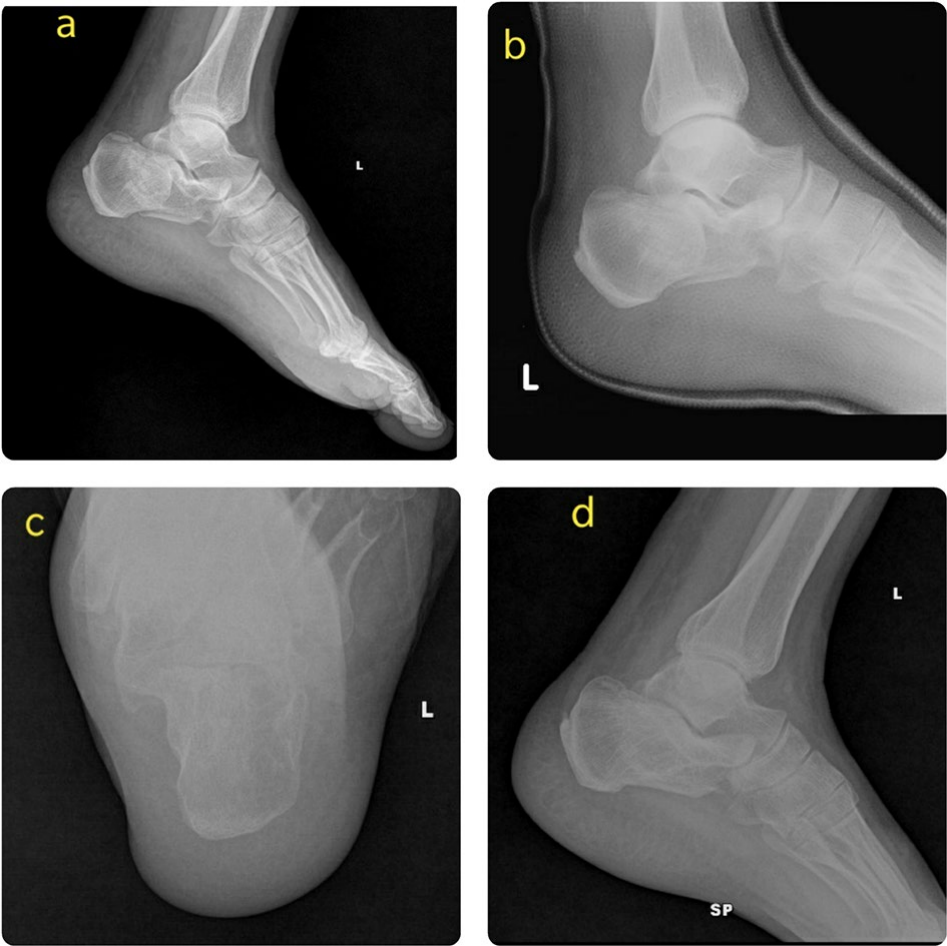
Calcaneal fracture treated by non-operative method. (**a**) Post injury radiograph, (**b**) radiograph after casting, (**c**,**d**) radiographs at one year follow up.

**Figure 2:**
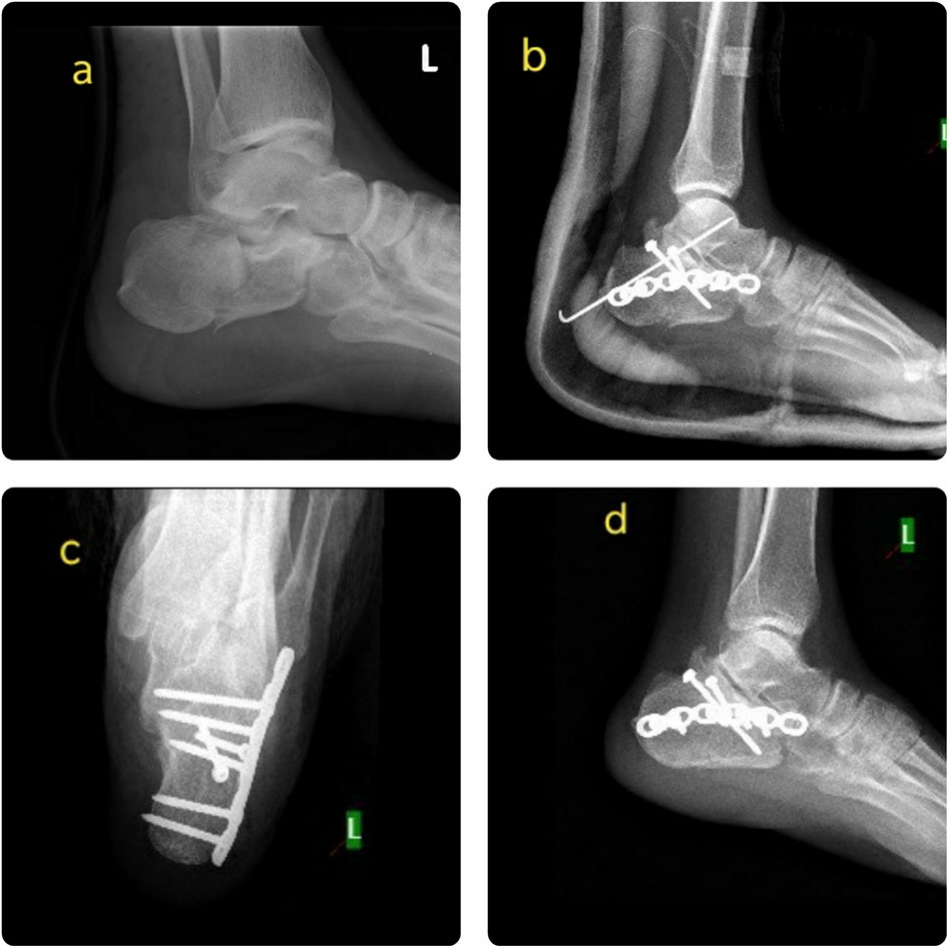
Calcaneal fracture treated by operative method. (**a**) Preoperative radiograph, (**b**) post-operative radiograph, (**c**,**d**) radiographs at one year follow up.

Pre and postoperative Bohler’s angle^5^ was calculated using MB ruler in the Computerized Radiographic system of our hospital. To avoid inter-observer bias, the values were checked by two medical assistants and a mean value was taken. Thirty calcaneal fractures were treated operatively and thirty-one non-operatively. Non-operative treatment was done with below-knee cast and non-weight bearing crutch walking for six weeks. After six weeks, the cast was removed and radiographs were done. Based on X-ray features, patients were gradually mobilized with partial weight-bearing as per their pain tolerance and full weight-bearing was started after four months (Fig. 1). Operative treatment was done with one of these methods—(i) percutaneous reduction and fixation with Essex-Lopresti maneuver/Cannulated Cancellous screws/K wires, (ii) Open reduction and internal fixation with Plates/Cannulated Cancellous screws/ K wires. Patients treated by operative method were put on splints with non-weight bearing mobilization for up to 4 weeks. From 4 to 8 weeks, active mobilization of toes and ankles were started. Gradual partial weight bearing was allowed from 8 weeks onwards with strengthening exercises and full weight bearing was allowed after 12 weeks (Fig. 2).

Patients were followed up for a minimum period of one year. The outcome measures were Modified Rowe’s Score (MRS)^6^, Visual Analogue Scale (VAS)^7^ and The American Orthopaedic Foot and Ankle Society (AOFAS)^8^ scale. The results of operative and non-operative groups were compared. Outcome related to the patient’s job at one year follow up was determined. Complications of treated calcaneal fractures were noted. The data was entered in MS Excel spreadsheet and statistical analysis was done using Statistical Package for Social Sciences (SPSS) version 16.0. Data analysis was done using the student’ unpaired T-test. A *p* value ≤ 0.05 was considered significant.

### Ethics approval

Institutional ethics committee clearance taken and all the guidelines and regulations followed.

## Results

All fifty-five patients were male. Age of the patients ranged from 18 to 65 years with a mean age in the non-operative group was 35 and the operative group was 34.9 years. Twenty-three patients had a fracture on the right side, twenty-six on the left side and six were bilateral. The majority of the calcaneal fractures were caused by fall from height (51 cases), two cases were due to slip and fall from stairs, 1 due to falling of a heavy object over the foot and 1 road traffic accident. Two patients had stable L1 compression fracture without deficits, 1 patient had distal radius fracture and 1 patient had fractures of the head of the radius and ulna styloid process. Forty-six patients were manual labourers, four were electricians, two were tree climbers and three were students. Thirty fractures were treated operatively and thirty-one non-operatively.

As per Sanders classification (Table 1), there were seven cases with type 4ABC, six with type 3BC, five with type 3AC, three with type 3AB, two with type 2C, six with type 2B and one with type 2A fracture in the operated group. In the conservative group, there were eight with type 4ABC, six with type 3BC, four with type 3AC, five with type 3AB, four with type 2C, two with type 2B and two with type 2A fracture.

**Table 1:** Fractures classified as per Sanders types and mode of treatment.

	Sanders classification							
	2A	2B	2C	3AB	3AC	3BC	4ABC	Total
Operative group	1	6	2	3	5	6	7	30
Non operative	2	2	4	5	4	6	8	31

Out of thirty treated operatively, eleven fractures underwent Essex Lopresti procedure ± CC screw fixation, nine fractures underwent percutaneous fixation of CC screw, six fractures underwent open reduction and internal fixation (ORIF) using CC screw and four underwent ORIF with plating. Thirty-one fractures were treated with cast immobilization.

Out of thirty Operated cases, Bohler’s angle was restored in twenty-five cases and showed good results with MRS at one year. The remaining five cases showed fair results. Thirty-one cases treated non-operatively showed fair results (Table 2).When compared at one year, operated cases showed better functional outcomes with MRS, VAS, and AOFAS (Table 3). Table 4 shows a number of complications encountered during this study. The outcome related to the job of the patient at a year follow up showed better results in the operative group (Table 5).

**Table 2:** Results of different Sanders types and treatment modalities.

Sanders type	Treatment given	No	Post op Bohler’s angle (18°–40°)	MRS score at 1 year
2A	Percutaneous CC screw	1	Within range	Good (85)
	Cast application	2	Not within range	Fair (65, 60)
2B	Essex Lopresti	3	Within range	Good 75,85,75
	ORIF with CC screw	1	Not within range	Fair 55
	Percutaneous CC screw	2	Within range	Good (85,75)
	Cast application	2	Not within range	Fair 45,50
2C	Essex Lopresti	1	Within range	Good 75,85
	Percutaneous CC screw	1	Not within range	Fair 65
	Cast application	4	Not within range	Fair (Mean 50)
3AB	Essex Lopresti	2	Within range	Good 85,75
	Percutaneous CC screw	1	Within range	Good 75
	Cast application	5	Not within range	Fair (Mean 55)
3AC	Essex Lopresti	2	Within range	Good 70,75
	ORIF with CC screw	2	Within range	Good 75,75
	Percutaneous CC screw	1	Within range	Good 70
	Cast application	4	Not within range	1 showed good result, 3 gave fair results
3BC	Essex Lopresti	3	Within range	Good 70,75,80
	Percutaneous CC screw	1	Not within range	Fair 45
	ORIF with CC screw	2	Within range	Good 75,70
	Cast application	6	Not within range	2 showed good results, 3 gave fair and 1 poor
4	Percutaneous CC screw	2	Within range	Good 70,70
	ORIF with CC screw	1	Within range	Good 75
	ORIF with plating	4	2 cases not within range	Good 70,70 Fair 55,60
	Cast application	8	Not Within range	2 had good results,5 fair and 1 poor

**Table 3:** Comparison between operated group and the non-operated group at 1 year of follow up.

Score	Treatment	N	Mean	SD	T test
MRS 1 year	Operated	30	74.783	11.229	5.807 *P* < 0.001
	Non operated	31	57.368	7.335	
AOFAS 1 year	Operated	30	78.783	9.582	2.728 *P* = 0.008
	Non operated	31	71.211	8.121	
VAS 1 year	Operated	30	3.348	1.369	4.387 *P* < 0.001
	Non operated	31	4.944	0.802

**Table 4:** Complications of treated calcaneal fractures.

Complications	Number	Operative group	Non operative group
Gait abnormality	21	8	13
Stiffness	22	12	10
Heel pain	24	10	14
Plaster sores	4	–	4
Wound infection	2	2	–
Wound dehiscence	1	1	–

**Table 5:** Outcome related to the job of patients at 1 year follow up.

Outcome related to patient’s job	Operative group	Non operative group
No restrictions	12	9
Some restriction on usual occupation	9	13
Change of job/substantial restrictions	5	4
Unable to work	2	1

## Discussion

The treatment of calcaneal fractures is controversial. Many studies have been reported but there is a lack of consensus. There are studies that have shown better outcomes with non-operative management^9^. Some authors have investigated operative management and got good results^10–12^.

Algren et al.^1^ in 2013 compared operative and conservative management and results obtained at one year were not significantly different statistically. However Agren et al.^13^, in a post-hoc analysis of their results (published 2014) found significantly better results in the subgroup of patients with anatomic reduction. Conservative management showed fair results in our study. This may be due to a lack of compliance with rehabilitation protocol or due to improper selection of patients for conservative management. The outcome of surgically managed cases in our study is comparable to the results in the study by Algren et al.^1^. In the present study, the outcome of surgically treated cases was better when the Bohler’s angle was restored to normal range (more than 18°). Buckley et al.^3^ also found that the results were equivalent between operative and non-operative groups and except in some operated cases, results were uniformly good. However patients undergoing non-operative treatment were 6 times as likely to require secondary subtalar fusion than patients treated operatively.

In our study, two cases with type 4 fracture showed good results as the Bohler’s angle was restored whereas the other two cases with Bohler’s angle not restored gave poor outcome. Two cases of type 4 fracture treated by ORIF with plating, one case each of type 2C and type 3BC treated with percutaneous CC screw and one case of type 2B treated by open reduction and CC screw fixation showed fair results. The common factor in all these cases was that Bohler’s angle was not restored above 18°.

**Figure 3:**
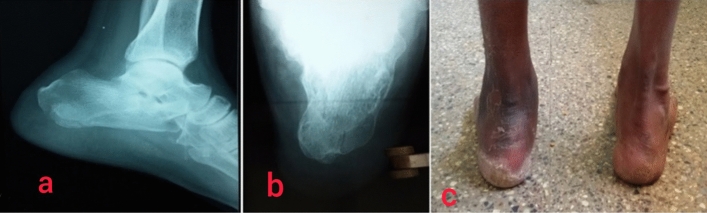
Complication after calcaneal fracture. (**a**,**b**) Radiographs showing calcaneal fracture with valgus deformation, (**c**) clinical photo of a patient with heel valgus deformity.

The complications were noted with both the treatment options. The commonest complications in both groups were stiffness, heel pain, and gait abnormalities (Fig. 3). The other complications like plaster sores and wound infection were specific to the conservative and operative group respectively. A study by Li et al.^14^ looked for the complication rate in the operated cases. In that study pain and necrosis were the commonest complications with the figures of 7.9% and 6.8% respectively. Infection, malunion, and loss of fixation were other complications encountered. The complication rate was 26.2% in the operated group and 13.7% in the non-operated group as per the study by Wei et al.^11^.

The present study also recorded the outcome related to the job of the patient at one year and compared with the pre-injury job status. While two patients in operated group and one in the conservative group were unable to work after one year, five patients in operated and 4 patients in the non-operative group had to change their job. Twenty-one patients in operated and twenty-two in the conservative group had no or minimal restrictions. Wei et al.^11^ found that the complication rate was higher with the operated group and also there was a significant number of patients who were unable to work at the final follow up.

The limitations of this study were sample size and lack of long term follow up. The results of our study need to be substantiated by a multicenter study with long term outcome assessments.

## Conclusion

Operative treatment of displaced intra-articular calcaneal fractures showed good results at one year when Bohler’s angle was restored to normal range. Non-operative treatment gave fair results. Complications were seen both with operative and non-operative treatment.
